# RETRACTION: Inhibiting of Proliferation, Migration, and Invasion in Lung Cancer Induced by Silencing Interferon‐Induced Transmembrane Protein 1 (IFITM1)

**DOI:** 10.1155/bmri/9852894

**Published:** 2025-10-24

**Authors:** BioMed Research International

RETRACTION: J. Yan, Y. Jiang, J. Lu, J. Wu, and M. Zhang, “Inhibiting of Proliferation, Migration, and Invasion in Lung Cancer Induced by Silencing Interferon‐Induced Transmembrane Protein 1 (IFITM1),” *BioMed Research International* 2019 (2019): 9085435, https://doi.org/10.1155/2019/9085435

The above article, published online on 08 May 2019 in Wiley Online Library (http://wileyonlinelibrary.com), has been retracted by agreement between the journal editorial board member, Salvatore Battaglia, and John Wiley & Sons Ltd. The retraction has been agreed due to figure concerns being raised on PubPeer [1]. On further investigation, further concerns were identified. Specifically:

• The left hand H460 panel in Figure 3d appears identical to the miR‐139 inhib panel in Figure 4e of [2].
• The righthand H460 panel in Figure 3d appear identical to the CDK‐14 siRNA panel in Figure 4e of [2].
• The GAPDH bands in Figure 1b appear similar to the GAPDH bands shown in Figure 2a and the B‐actin bands shown in Figure 2b of [3].
• The IFITM1 bands in Figure 1d appear similar to the USP22 bands shown in Figure 1d of [4].
• The Cyclin D1 bands in Figure 5a appear similar to the Cyclin D1 bands shown in Figure 4a of [5]
• The GAPDH bands in Figure 5a appear similar to the B‐actin bands shown in Figure 4a of [5]

The authors did not provide a response to the retraction.
